# On the Phylogenetic History of the Sanje Mangabey (*Cercocebus sanjei*), Udzungwa Mountains, Tanzania

**DOI:** 10.1007/s10764-026-00559-w

**Published:** 2026-04-27

**Authors:** Christina Lynette Paddock, Maria Joana Ferreira da Silva, Gráinne Michelle McCabe, David Fernández, William Scott McGraw, Michael William Bruford

**Affiliations:** 1 School of Biosciences, Cardiff University, Cardiff, UK; 2 Bristol Zoological Society, Bristol, Avon UK; 3 Wilder Institute, 1300 Zoo Road NE, Calgary, AB T2E 7V6 Canada; 4 Department of Anthropology & Archaeology, University of Calgary, Calgary, Canada; 5 Department of Anthropology, The Ohio State University, Columbus, OH USA

**Keywords:** Udzungwa Mountains, *Cercocebus*, Phylogeny, East Africa, Conservation management, Evolutionarily significant units

## Abstract

An accurate understanding of phylogeny, including intraspecific diversity, is key for defining conservation measures. The Sanje mangabey (*Cercocebus sanjei*) is an Endangered primate endemic to the Udzungwa Mountains in Tanzania that inhabits montane forest fragments and occurs in two populations—Mwanihana Forest and Uzungwa Scarp Nature Reserve—located 100 km apart. The phylogenetic position of the Sanje mangabey relative to other *Cercocebus* species remains unclear. We aimed to clarify the phylogeny of the Sanje mangabey and estimate its divergence time from other species. We reconstructed phylogenies using nuclear (CD4 and TSPY) and mitochondrial (COII and control region) sequences in noninvasive DNA sampled from both the populations (n = 88 sequences). We found that the Sanje mangabey grouped with the central/East African mangabeys (*C. galeritus*, *C. chrysogaster*, and *C. agilis*) and estimated that it diverged from the common ancestor ~2.34 million years ago (MYA; 95% Highest Probability Density (HPD) interval: 1.73–2.95 MYA). Results suggest that the two extant Sanje mangabey populations represent distinct lineages, diverging 0.77 MYA (95% HPD interval: 0.43–1.15 MYA). This divergence and isolation into two montane forest fragments may be attributed to increasing aridification in eastern Africa beginning ~2.6 MYA, the formation of physical barriers to dispersal and competition with other species. By confirming the evolutionary distinctiveness of the Sanje mangabey relative to other *Cercocebus* species, this study highlights the need for dedicated conservation management policies. Such policies should also consider the two populations as candidate evolutionary significant units. Further morphological, genetic, and behavioural studies are needed to assess the extent of reproductive isolation in Sanje mangabeys. Our study highlights the need to identify cryptic diversity within threatened species in order to improve their conservation.

**Supplementary Information:**

The online version contains supplementary material available at 10.1007/s10764-026-00559-w.

## Abstract in Portuguese

Uma compreensão das relações filogenéticas – incluindo a diversidade intraespecífica – é fundamental para definir medidas de conservação, particularmente de primatas pouco estudados e/ou ameaçados de extinção. O mangabei-de-sanje (*Cercocebus sanjei*) é um primata ameaçado de extinção endémico das Montanhas Udzungwa, na Tanzânia. Foram identificadas duas populações, que habitam fragmentos florestais de montanha, distanciados cerca de 100 km. A posição filogenética do mangabei-de-sanje em relação a outras espécies de *Cercocebus* permanece desconhecida, o que tem importantes implicações para a sua conservação. Aqui, pretendemos esclarecer a filogenia do mangabei-de-sanje e estimar seu tempo de divergência em relação a outras espécies. As filogenias foram reconstruídas usando sequências nucleares (CD4 e TSPY) e mitocondriais (COII e região controlo) usando amostras recolhidas de forma não invasiva na Floresta Mwanihana e na Reserva Natural da Escarpa de Uzungwa (N = 117 e 56 amostras, respectivamente). Os resultados mostram que o mangabei-de-sanje se agrupa com os mangabeis da África Central/Oriental – *C. galeritus*, *C. chrysogaster* e *C. agilis* – e estimou-se que tenha divergido do ancestral comum com estas espécies há cerca de 2,34 milhões de anos atrás (MAA; intervalo HPD de 95%: 1.73 a 2.95 MAA). As duas populações atuais de mangabei-de-sanje representam linhagens distintas, tendo divergido há 0.77 MAA (intervalo HPD de 95%: 0.43 a 1.15 MAA). Esta divergência e isolamento em dois fragmentos florestais de montanha podem ser atribuídos a: 1) aumento da aridificação na África Oriental, com início há cerca de 2.6 MAA; 2) competição com outras espécies; e 3) formação de barreiras físicas à dispersão. Ao confirmar a distinção evolutiva do *Cercocebus sanjei*, este estudo destaca a necessidade de 1) as políticas de gestão da conservação do mangabei-de-sanje serem consideradas ao nível da espécie, e 2) as duas populações serem geridas de forma independente como unidades evolutivamente significativas. *The translated abstract was not copy-edited by Springer Nature.

## Introduction

Taxonomy and systematics are important considerations in conservation activities because they can impact priorities at international, national and local levels (Groves, 2014). Published taxonomies often differ and may be inflated or conflated depending on the species definition used or methodological idiosyncrasies (Lawler, 2018). Depending on the taxonomic unit considered for conservation, these results can have profound implications for how conservation programs are implemented (Stanton *et al.*, 2019). For example, taxonomic frameworks that recognise sub-specific variation as biologically meaningful and elevate such units to species level would necessitate substantially greater conservation investment than frameworks that recognise a single species (Kingdon, 1997; Groves & Grubb, 2011). In contrast, recognizing a single species would risk undervaluing species richness and the loss of genetic diversity (Stanton *et al.*, 2019). An example is the African red colobus monkeys (*Piliocolobus* spp.), a group with a long history of taxonomic discord (Linder *et al.*, 2024; Oates & Ting, 2015). Lack of consensus over which forms of red colobus are evolutionary distinct has influenced how conservation priorities are established for the members of the genus (Linder *et al.*, 2024; Oates & Ting, 2015). Furthermore, taxonomic bias may lead to unbalanced allocation of resources with respect to the diversity being conserved (Lawler, 2018), while taxonomies based solely on morphology may obscure diversity which might be detectable at the molecular level (Hoelzel *et al.*, 2019). For these reasons, a deeper understanding of phylogenetic relationships and intraspecific genetic diversity is key for informing conservation measures.

The concept of the evolutionarily significant unit (ESU) as originally developed intended to capture the breadth of diversity below the species level using a range of biological and genetic criteria (Ryder, 1986, Hoelzel, 2023). The ESU concept aimed to provide a theoretical background for prioritising taxa for conservation purposes, for instance in the face of economic constraints and the inability of taxonomy to reflect apparent genetic diversity (Ryder, 1986; Moritz, 1994). The objective of the ESU metric is to identify independently evolving populations to manage them as distinct units, which would preserve their evolutionary potential and promote the long-term persistence of species facing environmental changes (Ferreira da Silva & Bruford, 2017; Hoelzel, 2023). Numerous methodological frameworks have been proposed for identifying ESUs since its conception (Hoelzel, 2023), including the widely applied that defined ESU as populations that 1) are reciprocally monophyletic for mitochondrial DNA alleles, and 2) that demonstrate significant divergence of allele frequencies at nuclear loci (Moritz, 1994).

**Fig. 1:**
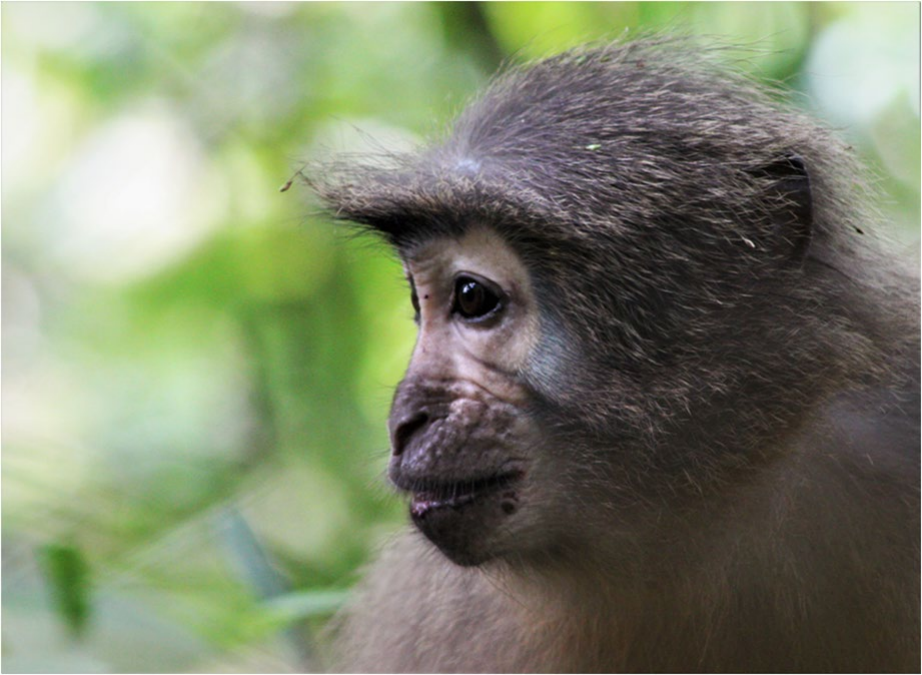
Side profile of the Sanje mangabey (*Cercocebus sanjei*) in Udzungwa Mountains, Tanzania, obtained during sampling in 2017, showing the white eyelids and backswept crest that resemble *C. galeritus,* and the distinctive beige facial colouring that differs from neighbouring *Cercocebus* populations. Photo credit by C.P.

**Fig. 2:**
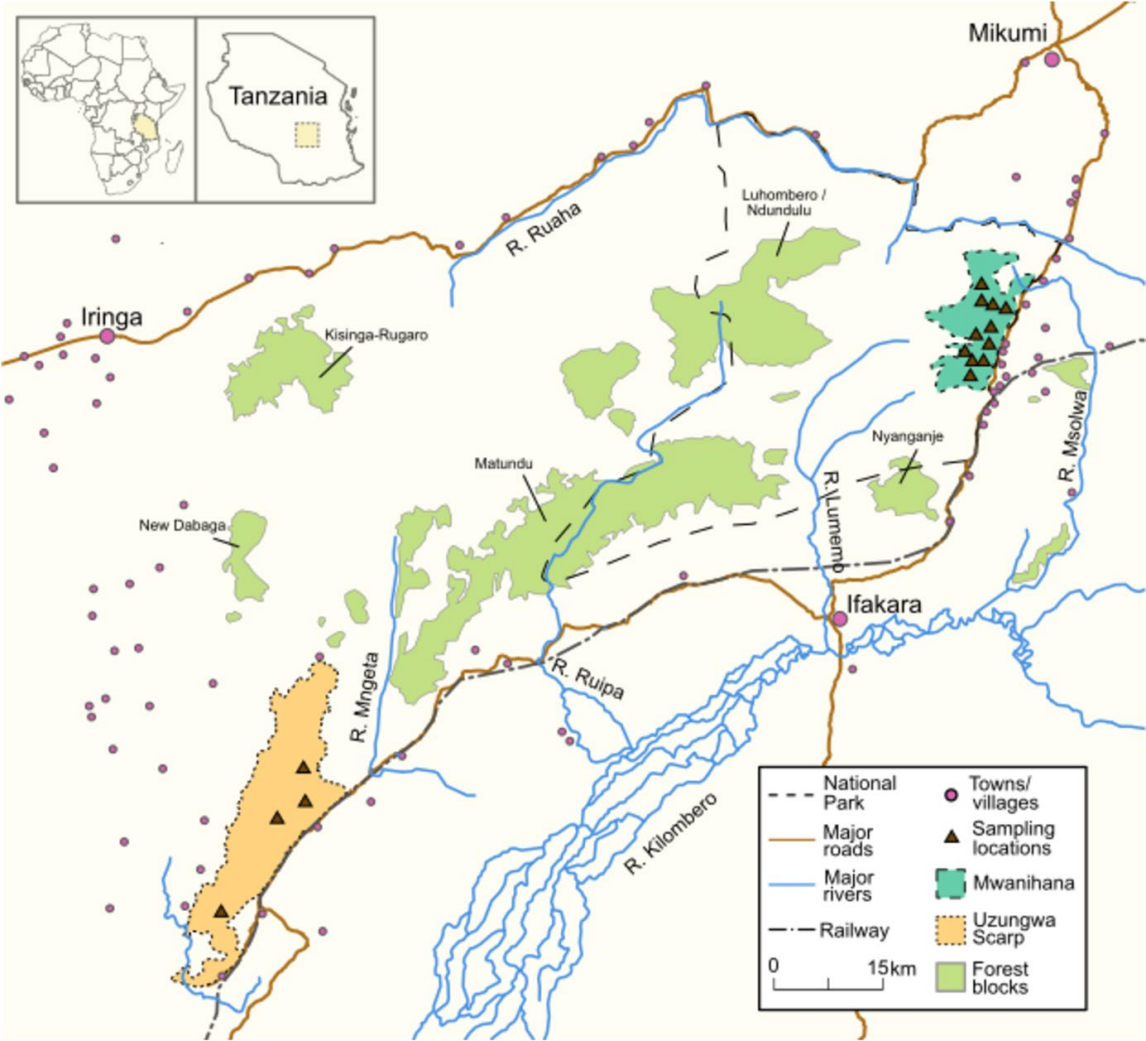
Map of the Udzungwa Mountains, Tanzania, with the forests Mwanihana (green; northeast) in the Udzungwa Mountains National Park boundary (dashed line) and Uzungwa Scarp Nature Reserve (orange; south-west) highlighted as the two forest blocks in which the Sanje mangabey (*Cercocebus sanjei*) is found. Sampling locations for faecal samples collected in this study between June and November 2017 are also marked.

**Fig. 3:**
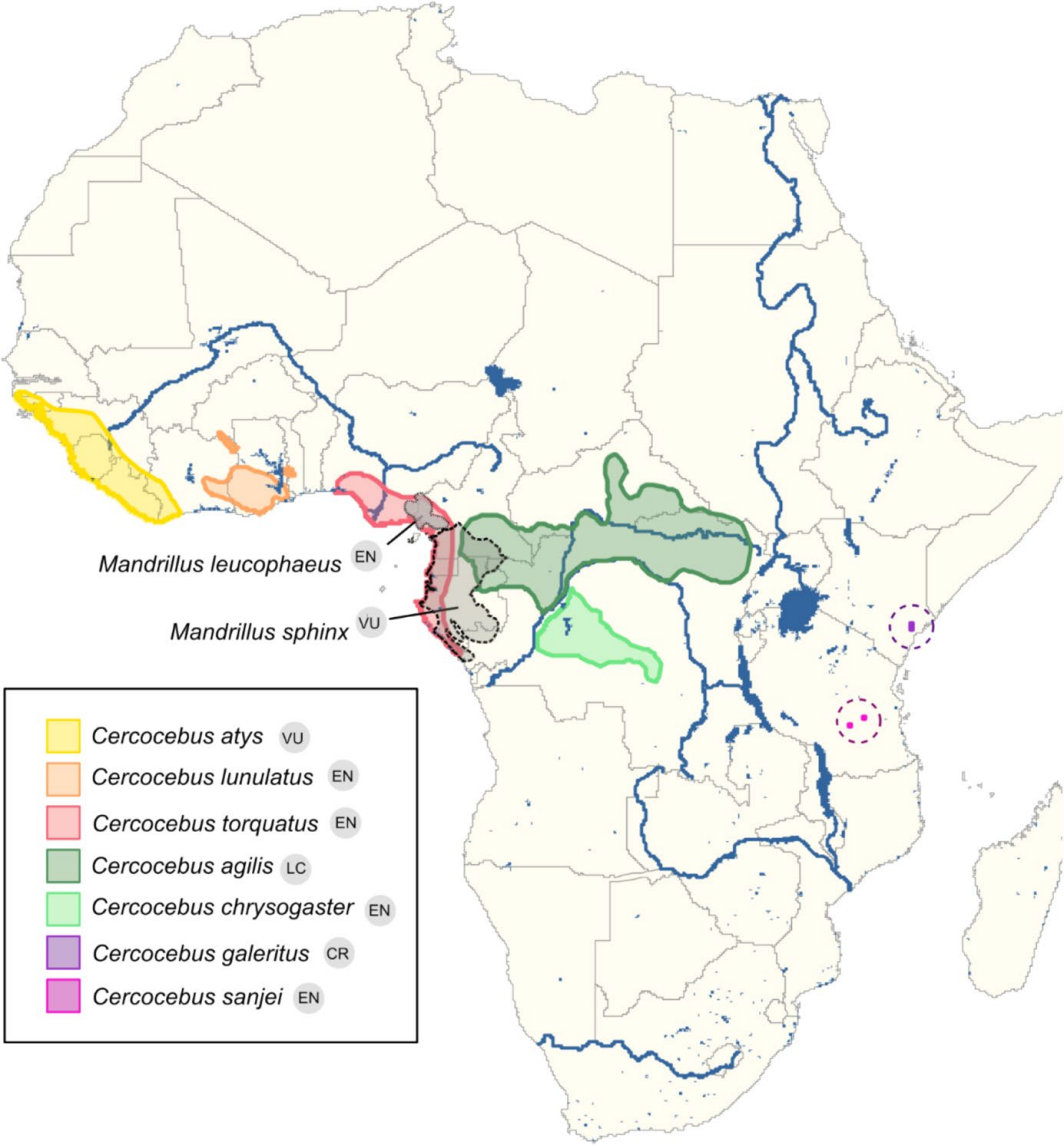
Distribution of the *Cercocebus* and *Mandrillus* species throughout Africa, with the distribution polygons downloaded from the International Union for Conservation of Nature (IUCN) Red List of the seven *Cercocebus* species in colour blocks and the two *Mandrillus* species outlined in greyed areas. The distribution of *C. galeritus* and *C. sanjei* are highlighted with a dashed circle owing to the small distribution range of each species. The IUCN conservation status of each species is included in the grey circles: Least Concern (LC), Vulnerable (VU), Endangered (EN), and Critically Endangered (CR). All species have a decreasing tendency of the population size (https://www.iucnredlist.org; downloaded on 18 December 2020).

The Sanje mangabey (*Cercocebus sanjei*; Mittermeier, 1986; Fig. 1) is a papionin primate endemic to the Udzungwa Mountains, Tanzania (McCabe *et al.*, 2019; Figs. 2). The Sanje mangabey has been included in the clade of the "*Mangadrills*", which includes all known *Cercocebus* and *Mandrillus* species (Fernández et al., 2019). The *Mangadrills* are distributed throughout west, central and east Africa and comprise nine species; seven *Cercocebus *and two *Mandrillus* (Fig. 3). The Sanje mangabey was originally considered a subspecies of *Cercocebus galeritus* (*C. galeritus sanjei*, Homewood & Rodgers, 1981) along with the Tana River mangabey (*C. galeritus galeritus*), the agile mangabey (*C. galeritus agilis*), and the golden-bellied mangabey (*C. galeritus chrysogaster*) (Supplementary material, Table S1). At that time, the Sanje mangabey was described as closely resembling other *C. galeritus* subspecies in terms of size, sexual dimorphism, and several individual features, including ischial callosity shape (Homewood & Rodgers, 1981). In frontal view, several features of the Sanje mangabey face (e.g., hair around the brow) resemble those present in the agile mangabey; however, in lateral view, the Sanje’s backswept crest, and white eyelids are more similar to the Tana River mangabey (Homewood & Rodgers, 1981) (Fig. 1). The Sanje mangabey pelage was described as “intermediate” between the Tana River mangabey and the agile mangabey; however, the Sanje mangabey differs from the Tana River mangabey*,* the agile mangabey and the golden-bellied mangabey in that its face colour is beige (Fig. 1), rather than dark grey/black (Homewood & Rodgers, 1981). Furthermore, the “whoop-gobble” vocalisations made by the Sanje mangabey*,* the agile mangabey and the Tana River mangabey seem indistinguishable, providing further support for considering these taxa subspecies of *C. galeritus* (Grubb *et al.*, 2003)*.* Based on its current distribution and hypothesized dispersal options, Homewood & Rodgers (1981) suggested the Sanje mangabey may represent an intermediate form between the agile mangabey and the Tana River mangabey. Since these studies, Mittermeier *et al.*, (2013) suggested that the Tana River mangabey, the agile mangabey, and the golden-bellied mangabey each should be afforded species status. Furthermore, several taxonomic authorities have argued that the Sanje mangabey is sufficiently distinct morphologically to be upgraded to full species status (Groves, 2001; Kingdon, 1997), but little evidence has been offered to support the latter, and the taxonomy of this group remains unresolved.

The Sanje mangabey has not been the subject of detailed morphological analysis due to a lack of monotype or other museum specimens, nor has it been included in broader studies of *Cercocebus-Mandrillus* phylogeny (Wieczkowski & Butynski, 2013), because no genetic sequence data are available (Supplementary material Section 1, Fig. S1). Thus, the relative position and lineage distinctiveness of the Sanje mangabey in the clade remains unknown (Devreese & Gilbert, 2015; Dempsey *et al.*, 2024).

The Sanje mangabey was first described by scientists in the early 1980 s (Homewood & Rodgers, 1981) and has been listed as Endangered by the International Union for Conservation of Nature (IUCN) Red List since 1988 (McCabe *et al.*, 2019). Forest fragmentation, habitat degradation, and illegal hunting are identified as its primary conservation threats (Oberosler *et al.*, 2020). The recently published *Cercocebus-Mandrillus* Conservation Action Plan (Dempsey *et al.*, 2024), recommends five actions for all species in the clade: (i) Increase stakeholder engagement; (ii) Habitat restoration; (iii) Elevate the profile of all species; (iv) Enhance protection; and (v) Reduce existing knowledge gaps. Several unresolved taxonomic issues in the clade, including those specific to the Sanje mangabey (Dempsey *et al.*, 2024), need to be addressed to maximize effectiveness of conservation management.

The Sanje mangabey is currently distributed in two populations inhabiting forest fragments, the Mwanihana and the Uzungwa Scarp forests, which are approximately 100 km apart (Fig. 2, Fig. 3). The Sanje mangabey is generally considered forest-adapted (Ehardt *et al.*, 2005) and is likely unable to disperse across the drier habitats surrounding Mwanihana and the Uzungwa Scarp forests. We do not know when the populations and forest blocks across the Udzungwa Mountains became separated (Struhsaker *et al.*, 2004), whether the two populations are evolutionarily distinct, or if they should be managed as separate conservation units (Dempsey *et al.*, 2024). One priority for the Sanje mangabey in the *Cercocebus-Mandrillus Conservation Action Plan* was to clarify the taxonomic and evolutionary relationships between the populations (Dempsey *et al.*, 2024). Studies of other species in the Udzungwa Mountains have indicated significant divergence between subpopulations in the Mwanihana and Uzungwa Scarp forest fragments (Bowkett *et al.*, 2015; Ceccarelli *et al.*, 2014; Ruiz-Lopez *et al.*, 2016).

We investigated the phylogenetic history of the Sanje mangabey using molecular data with the overarching goal of informing and improving the conservation management of its two remaining populations, following recommended actions in the *Cercocebus-Mandrillus Conservation Action Plan*. This study has three aims: 1) to determine the phylogenetic position of the Sanje mangabey in the *Cercocebus-Mandrillus* clade; 2) to assess whether the Sanje mangabey represents a distinct lineage relative to other *Cercocebus* species and estimate the date of the most recent common ancestor; and 3) to investigate the genetic distinctiveness of the two Sanje mangabey populations. We estimated the phylogenetic relationship between the Sanje mangabey and other *Cercocebus* mangabeys by using two nuclear (autosomal CD4 gene and Y chromosomal testis-specific protein TSPY), and two mitochondrial fragments (cytochrome oxidase subunit II: COII; and control region: CR). The fragments represented three inheritance pathways: mitochondrial maternal inheritance (COII and CR), Y-chromosome inheritance (TSPY), and nuclear autosomal inheritance (CD4). Each pathway can influence the genetic structure of populations differently depending on the life history and dispersal behavior of a species, so we compared phylogenies constructed by using each inheritance pathway to identify differences in clustering patterns.

## Methods

We collected fecal samples noninvasively from unidentified individuals between June and November 2017 in the two forests in the Udzungwa Mountains, Tanzania, where the Sanje mangabey is endemic: (i) Mwanihana Forest (7°40’−7°57’S, 36°46’−36°56’E) in Udzungwa Mountains National Park, and (ii) Uzungwa Scarp Nature Reserve (8°14’−8°32’S, 35°51’−36°02’E; Fig. 2).

We searched for mangabeys in 28 locations (13 in Mwanihana and 15 in Uzungwa Scarp; Fig. 2) at the same time as an acoustic population survey (Paddock *et al.*, 2020). To locate groups, trained observers climbed to vantage points to locate the distinctive mangabey “whoop-gobble” vocalisation. When observers detected a group, they observed it from a distance and moved towards the site to collect fecal samples after the mangabeys left. We could not characterize the groups sampled in terms of group size or sex ratio due to low visibility. We collected fecal samples only if they were 2 m apart to minimise the chance of resampling individuals. We recorded the location and elevation of sampling sites using Geographic Positioning System (GPS) coordinates (Garmin GPSMAP 54 s Handheld Navigator device).

We collected feces whole and stored them using the “two-step method” (Roeder *et al.*, 2004). We immersed the sample in 30 ml of 97% ethanol in a sterile 25-ml universal falcon tube (SARSTEDT AG & Co., Nümbrecht, Germany) for 48 hr. Then, we drained the ethanol and replaced it with approximately 10 g of Silica Orange (Sigma-Aldrich® Company Ltd., Dorset, UK). We stored the samples in the silica gel at room temperature until DNA extraction.

We performed DNA extraction in the School of Biosciences, Cardiff University, UK. We scraped and collected the outermost surface of the feces using a scalpel as we expected this material to contain the highest amount of host DNA and the lowest concentration of PCR inhibitors from the diet (Beja-Pereira *et al.*, 2009). We extracted DNA using the QIAamp Fast DNA Stool Mini Kit (Qiagen, UK) and stored extracts at −20 °C until required for analyses. We minimised the risk of contamination from exogenous DNA by conducting all DNA extractions in a laminar-flow hood in a different laboratory to the PCR setup and sterilized all surfaces with bleach and ethanol prior to work. When handling samples in collection and processing, we wore gloves, face masks, and plastic head caps to prevent contamination with exogenous DNA and to minimise the risk of zoonoses.

We chose the fragments analysed to include freely available sequences produced in previous phylogenetic studies of Papionini (Burrell *et al.*, 2009; Davenport *et al.*, 2006; Disotell *et al.*, 1992; Harris, 2000; Harris & Disotell, 1998; Olson *et al.*, 2008; Tosi *et al.*, 2003; Zinner *et al.*, 2009). Because no publicly available sequence data existed for the Sanje mangabey, we designed primers using conserved regions in alignments of *Cercocebus* sequences obtained from GenBank (Supplementary material Table S2). We targeted fragments using two to three overlapping primers (Table I).

**Table I:** Primer sequences used to amplify fragments of two nuclear loci; CD4 and TSPY, and two mitochondrial genes; control region (CR) and cytochrome oxidase subunit II (COII) in the Sanje mangabey (*Cercocebus sanjei*) Udzungwa Mountains, Tanzania (data collected between June and November 2017)

Region	Primer	Sequence (5’ – 3’)	Fragment length (bp)	PCR cycling conditions
CR	CRF-1	GCTCCGGGCCCATAACTC	257	95 °C for 2 min, [95 °C for 30 s, 61.8 °C for 60 s, 72 °C for 2 min] x 35 cycles, 72 °C for 10 min
	CRR-1	CAAAGACAGGCGCATTCAGG		
	CRF-2	CCRAAACATGCTTACAAGC	343	95 °C for 2 min, [95 °C for 30 s, 50 °C for 60 s, 72 °C for 2 min] x 35 cycles, 72 °C for 10 min
	CRR-2	GTTATGGCCCTGAGGTAAG		
COII	COIIF-1	CTATATGCCCTRTTCTCAAC	297	95 °C for 15 min, [94 °C for 30 s, 50 °C for 90 s, 72 °C for 90 s] x 40 cycles, 72 °C for 10 min
	COIIR-1	CTTCTAGGAGTCGAAGGTC		
	COIIF-2	GACYAYGGAGGCCTAATC	303	
	COIIR-2	GTTCYGCRACGATTGG		
TSPY	TSPYF-1	CAGTTGAGAGGTGCTCTTG	264	
	TSPYR-1	CACAGTCCCTTAACAATAGC		
	TSPYF-2	CTGAAGAGCAGAAGCGAG	324	95 °C for 15 min, [94 °C for 30 s, annealing for 90 s, 72 °C for 90 s] x 20 cycles with annealing starting at 64 °C and decreasing by 1 °C every 2 cycles, [94 °C for 30 s, 54 °C for 90 s, 72 °C for 90 s] x 20 cycles, 72 °C for 10 min
	TSPYR-2	GGAAGGCCTAAGAGCACC		
	TSPYF-3	CTCAGACACCGGCAGTTC	290	
	TSPYR-3	CATCTTGGTCAGTGATCAGG		
CD4	CD4F-1	CCAAATCCAGCCTGAGCTG	253	
	CD4R-1	CAGCCAAGACAGGGTTTCC		
	CD4F-2	CTGTCAAACTGGCCTCCG	351	
	CD4R-2	GAGTTGGCAGTCACTGTGG		

We targeted a total fragment size of 401 base pairs (bp) of CD4 (spanning nucleotide positions 127–527 in *Cercocebus* *atys* AF057383) by amplifying two overlapping fragments of 253 and 351 base pairs (bp) in length by polymerase chain reaction (PCR). To design two primer pairs in conserved regions, we aligned four *Cercocebus* sequences (*C. agilis*: FJ750597; *C. chrysogaster*: AF057382; and *C. atys:* AF057383; AF057384). For TSPY, we targeted a total fragment size of 621 bp (spanning nucleotide positions 113–733 in *C. galeritus* AY195576) by overlapping sequences amplified by three primer pairs. We designed primers in conserved regions of nine *Cercocebus* sequences after aligning sequences from GenBank (*C. agilis*: FJ750633; *C. galeritus*: AY195576; *C. chrysogaster*: AF057409 and AF057410; *C. atys*: AF057411, AF057412, AF057413, and AF057414; and *C. torquatus*: AY195577). We targeted a 523 bp fragment for COII region (spanning nucleotide positions 7,146–7,647 in *C. galeritus* M74004) using overlapping sequences from two primer pairs, which we designed in conserved regions of an alignment of *Cercocebus* sequences from GenBank (*C. agilis*: FJ750650; *C. galeritus*: AY686132 and M74004; *C. atys*: AY686135; *C. torquatus*: FJ713422 and AY686133; and *C. lunulatus*: AY686134). We designed two primer pairs for mtDNA Control Region (CR) in conserved regions of three *Cercocebus* mitochondrial genomes (*C. torquatus*: NC_023964; and *C. atys*: NC_028592 and KP090062), with the overlapping sequence 515 bp in length (spanning nucleotide positions 15,648-16,218 in *C. atys* NC_028592).

We amplified each fragment by PCR in a total volume of 10 μl, consisting of 5 μl of QIAGEN Multiplex PCR Master Mix (for CD4, TSPY and mtDNA COII) or 5 μl of Bioline MyTaq™ (for mtDNA CR), 1 μl template DNA, and forward and reverse primers at a final concentration of 2 μM. We ran all PCR cycling programmes (Table I) in an Applied Biosystems GeneAmp PCR System 9700. We tested the success of each PCR by using agarose gel electrophoresis visualized in a UV transilluminator. We sequenced successful PCR amplifications bi-directionally by using Sanger technology at Eurofins PlateSeq Service (Ebersberg, Germany) and Centre for Molecular Analysis sequencing service (CTM; CIBIO, Portugal).

We inspected the sequence chromatograms by eye using Geneious (v4.8.5). We assessed the sequences for nuclear copies of mitochondrial DNA (NUMTS) following recommendations from Bensasson *et al.* (2001). Specifically, we screened the chromatograms for double electrophoretic peaks and removed all sequences of lower quality or showing double electrophoretic peaks from the final dataset.

We used a 64 CR sequences (36 from Mwanihana Forest and 28 from Uzungwa Scarp Nature Reserve) obtained from a study of the phylogeographic structure of Sanje mangabey subpopulations (Paddock *et al.*, 2026). We included each unique haplotype sequence found in Paddock *et al.*, 2026) in this study.

We submitted each sequence to BLAST (Basic Local Alignment Search Tool; https://blast.ncbi.nlm.nih.gov/Blast.cgi) to search against existing sequences in GenBank for the genetically closest sequence and species. We only included samples where the top results for percentage identity (>95%) were *Cercocebus* sequences in further analyses.

To construct phylogenies of the Sanje mangabey within the Papionini tribe, we retrieved sequences from GenBank for *Cercocebus, Mandrillus, Lophocebus, Rungwecebus, Theropithecus,* and *Papio* species. We chose *Macaca* as an outgroup (accession numbers of sequences used in Supplementary material Table S2). We generated sequences from each forest and included them in separate datasets. We aligned unique haplotypes from each subpopulation for CD4, TSPY, and COII and CR fragment to sequences for other species. We trimmed all sequences to the length of the smallest sequence to reduce missing data between samples, resulting in an alignment of 400 bp for CD4 (spanning nucleotide positions 126–525 in *C. chrysogaster*: AF057382), 588 bp for TSPY (spanning nucleotide positions 113–700 in *C. galeritus*: AY195576), 480 bp for COII (spanning nucleotide positions 7,168-7,647 in *C. atys*: NC028592), and 369 bp for CR (spanning nucleotide positions 15,689–16,057 in *C. atys*: NC028592).

We constructed Maximum Likelihood and Bayesian phylogenies. These methods can show discrepancies in branch support estimates (i.e., Maximum Likelihood in bootstrap and Bayesian methods in posterior probability estimates) and can be used to estimate upper and lower bounds of support for branches (Douady *et al.*, 2003). We reconstructed three phylogenies with each method using partitioned concatenated sequences: 1) concatenating the two mitochondrial fragments (CR/COII; 849 bp); 2) concatenating the two nuclear markers (TSPY/CD4; 988 bp); and 3) all four fragments concatenated (CR/COII/TSPY/CD4; 1,833 bp). To select the most appropriate nucleotide substitution model for each fragment, we used jModelTest v2.1.10 and the Bayesian Information Criteria (BIC) to determine the substitution model for both Maximum Likelihood and Bayesian phylogeny reconstruction (CD4 = HKY; CR = HKY + G; COII = HKY + G; TSPY = JC). We reconstructed Maximum Likelihood phylogenies in RAxML v8.2.10 (Stamatakis, 2014) with 1,000 bootstrap replicates and generating an extended majority rule tree with a >50% threshold from these replicates. We assigned the *Macaca* sequence in each alignment as the outgroup. We generated a Bayesian phylogeny with posterior probabilities using MrBayes 3.2.7 (Ronquist *et al.*, 2012) and a Monte Carlo Markov Chain (MCMC) with 1,000,000 generations, sampling every 1,000 and with a 25% burn-in. We evaluated convergence of models using the potential scale reduction factor (PSRF ~1.0) and effective sample size (ESS >100) for all parameters.

To investigate the time to the most recent common ancestor (TMRCA) for *Cercocebus* species, we created a calibrated phylogeny for the concatenated sequence of all fragments (CD4-TSPY-COII-CR; 1,837 bp fragment) using BEAST2 v2.6.0 (Bouckaert *et al.*, 2019). We defined each fragment as a separate partition with the most appropriate substitution model as estimated for each fragment in the above Maximum Likelihood and Bayesian phylogenies. We selected individual site and clock models. We constructed the divergence tree by using a Calibrated Yule Model, which is appropriate for comparing sequences from different species and previously published Papionini divergence time estimates as priors to calibrate the model from the phylogenetic history study of the kipunji (*Rungwecebus kipunji*; Zinner *et al.*, 2009; Table II). We included a divergence time between *Cercocebus* and *Mandrillus*, enforcing monophyly, following strong statistical support (Devreese & Gilbert, 2015) and a study of divergence within Papionins (Zinner *et al.*, 2009). To calibrate the model, we used an estimate of the divergence time between *Papio* and *Theropithecus* from the fossil record (3.5–4 MYA; Benefit, 2002; Delson *et al.*, 2000; Leakey, 1992; Table II). We used a normal distribution to set the priors across the 95% confidence interval.

**Table II:** Divergence time estimates and confidence interval used as priors to calibrate a Calibrated Yule Model for the Sanje mangabey (*Cercocebus sanjei*) within the Papionini tribe

Papionini tribe species			Divergence estimate (MYA; 95% CI)	Reference
*Papio*	/	*Theropithecus*	3.75 (3.50–4.00) (Fossil)	Delson *et al.*, 2000; Leakey, 1992
*Macaca*	/	*Lophocebus, Theropithecus, Rungwecebus, Papio, Mandrillus* &* Cercocebus*	9.18 (7.71–10.48)	Zinner *et al.*, 2009
*Lophocebus*	/	*Theropithecus, Rungwecebus* & *Papio*	4.95 (3.79–6.15)	
*Cercocebus* & *Mandrillus*	/	*Theropithecus, Rungwecebus, Lophocebus* & *Papio*	8.47 (6.98–10)	
*Cercocebus*	/	*Mandrillus*	3.73 (2.09–5.68)	

Selecting appropriate calibration points is essential for obtaining meaningful estimates of divergence times (Graur & Martin, 2004). The use of secondary calibrations points as the only source of calibration in divergence time analyses can increase the error in the date estimates (Schenk, 2016). Using multiple fossil calibration points within the clade of interest, that are both reliable and independent, yields robust results (Raaum *et al.*, 2005). However, this remains challenging due to the incompleteness of the fossil record and the inherent difficulties in interpreting it. We used an estimate of the divergence time between *Papio* and *Theropithecus*, because these were the taxa most closely related that have a well-dated split since *Theropithecus* appears in the fossil record by 3.5–4 MYA (Delson *et al.*, 2000; Leakey, 1992). Moreover, we used three of four fragments (CD4, TSPY, and COII), which were used in a study that estimated the position of kipunji in the Papionini tribe (Zinner *et al.*, 2009) and included the divergence time estimates in calibrating the model in our study.

We ran the model as four independent replicates for 10 million steps, sampling every 1,000 steps with a 10% burn-in, and combined runs by using LogCombiner v2.5.2. We inspected the models in TRACER v1.7.1 (Rambaut *et al.*, 2018) and refined priors until ESS values were >200. We summarised the combined replicates (40,004 trees processed) generated by the model in TreeAnnotator v2.5.2 using default parameters and visualised them in FigTree v1.4.4 (Rambaut, 2018).

## Ethical Statement

Our research complied with rules and protocols approved by national organizations and adhered to the legal requirements of the United Republic of Tanzania. Tanzania National Parks, Tanzania Forest Service Agency, Commission for Science and Technology, and the Tanzanian Wildlife and Research Institute, approved the study conducted in Tanzania. We obtained fecal samples non-invasively from unidentified individuals without manipulation or perturbation of their behavior. DEFRA (APHA) authorized the importation of the samples to the UK (Authorisation No: ITIMP17.0097, issued on 30/01/2017).

### Data Availability

DNA sequences produced during this work have been deposited on the GenBank database with accession numbers PP896812 to PP896819. Additional data are available in the Supplementary Information.

## Results

We collected and analysed 173 fecal samples; 117 samples in Mwanihana Forest and 56 samples in Uzungwa Scarp Nature Reserve (Fig. 2). We obtained 88 sequences of good quality across all markers (Supplementary material Table S3, Mwanihana Forest: n = 46, Uzungwa Scarp: n = 42, 51% sequencing success). Sequencing success varied among fragments, with mitochondrial CR showing the highest (64 sequences) and nuclear markers the lowest (6 sequences each) success.

We found no evidence for the presence of NUMTs, such as double electrophoretic peaks in the chromatograms, in sequences considered of good quality and used for analyses. After visualization of the chromatograms, we excluded low-quality sequences from subsequent analyses. We also removed ten sequences from further analyses following submission to BLAST (Mwanihana Forest: n = 3; Uzungwa Scarp: n = 7), because they showed >95 percentage identity with existing *Cercopithecus mitis* GenBank database sequences, suggesting that these samples were from Sykes monkeys that live sympatrically with the two Sanje mangabey populations.

The two nuclear fragments we used, autosomal CD4 gene (CD4; 400 base pairs [bp]) and Y chromosomal testis-specific protein (TSPY; 588 bp), showed very low genetic variation and hence differentiation between the two Sanje mangabey populations. The CD4 marker had only one segregating site and the alignment showed a mean of 99.0% (SD ±0.60) pairwise identity between all species and 97% identical sites between sequences. CD4 showed one segregating site between forests (2 unique sequences overall). The TSPY sequence was identical in the two Sanje mangabey populations and showed a 98.7% (SD ±0.64) pairwise identity between all species and 94.4% identical sites between sequences.

The mitochondrial (mtDNA) cytochrome oxidase subunit II (COII; 480 bp) fragment was identical between Sanje mangabey populations. When comparing species, it showed a 91.2% (SD ±4.15) mean pairwise identity and 71.9% identical sites between sequences. The mitochondrial control region (CR; 369 bp) fragment was the most variable sequence studied, with 22 segregating sites between Sanje mangabey populations and a mean 84.7% (SD ±7.76) pairwise identity between all *Cercocebus* species, with 64.2% identical sites between sequences.

### Phylogenetic Reconstruction

**Fig. 4:**
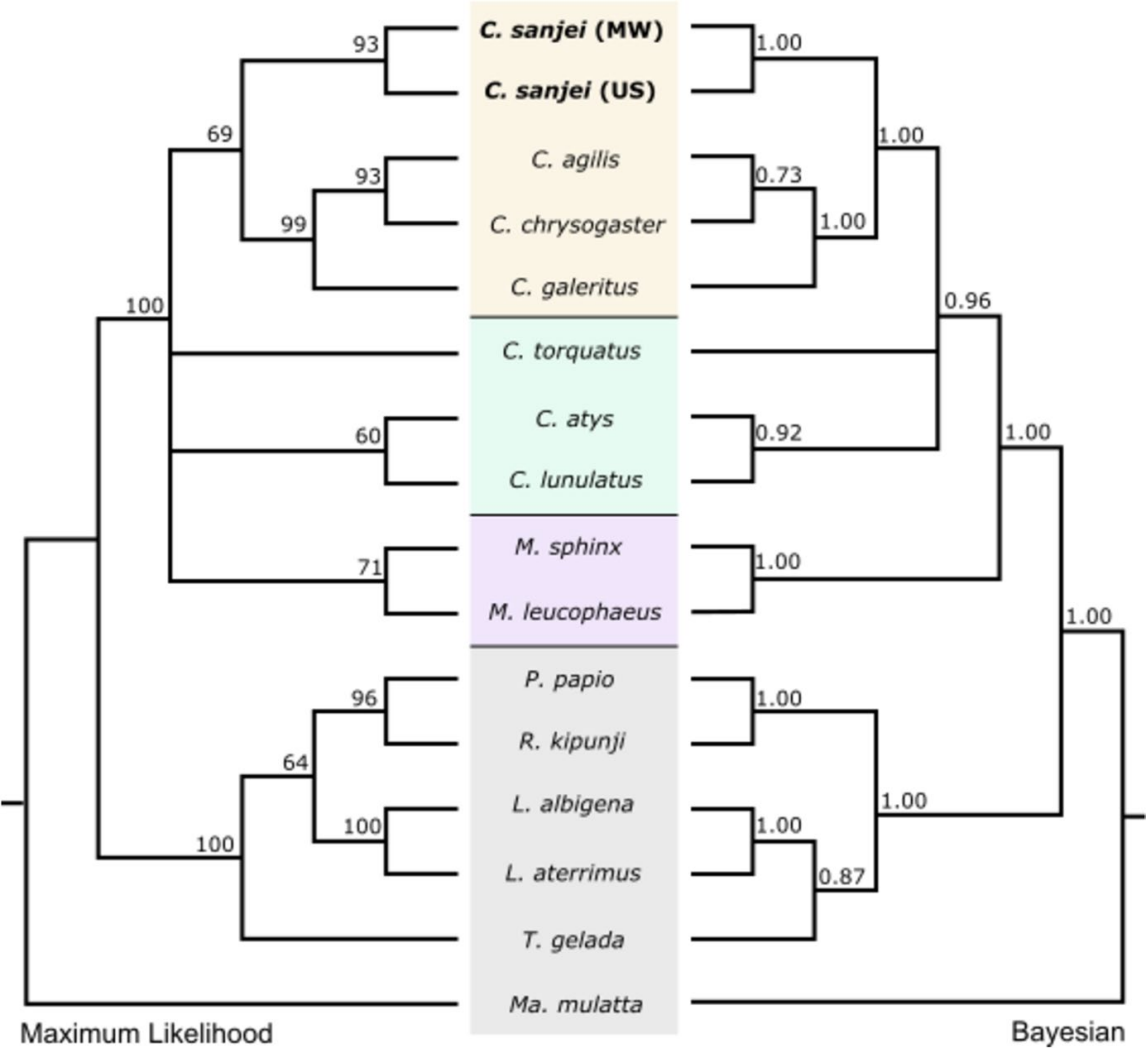
Phylogeny of the Sanje mangabey (*Cercocebus sanjei*), Udzungwa Mountains, Tanzania (data collected between June and November 2017). Phylogenetic trees for papionin species using a concatenated sequence (1,833 base pairs) for four fragments: two nuclear (CD4 and TSPY) and two mitochondrial (COII and CR). Both the maximum likelihood phylogeny (left; bootstrap values shown at the nodes) and the Bayesian phylogeny (right; posterior probabilities shown at the nodes) are presented. Sequences used in the phylogeny are available in Table S2, supplementary material. *Cercocebus* species in Central/East Africa, each previously considered subspecies of *Cercocebus galeritus,* are coloured in yellow; the remaining *Cercocebus* species, found in Central/West Africa, are coloured green; *Mandrillus* species coloured purple; and other papionin species coloured in grey.

The three phylogenies positioned the Sanje mangabey in a group that included all other *Cercocebus* species with high or relatively high Posterior Probability (PP) and percentage of bootstrap value (BV, %), using both Bayesian and Maximum Likelihood approach for each phylogeny (six total reconstructions) (Fig. 4; Supplementary material Figs. S2a and b). The PP and BV for each phylogeny were 1) PP = 1.00 and BV = 100% considering all four fragments (CD4, TSPY, COII, and CR, concatenated, 1,833 bp; Fig. 4); 2) PP = 1.00 and BV = 98%; considering COII and CR mitochondrial fragments concatenated, 849 bp, Supplementary material, Fig. S2a); and 3) PP = 1.00, BV = 100% considering CD4 and TSPY nuclear fragments concatenated (988 bp, Supplementary material Fig. S2b). The sequences used in each reconstruction are available in Supplementary material Table S2.

Phylogenies produced using the concatenation of all four fragments showed relatively high support for the grouping of *Cercocebus sanjei* with *C. agilis*, *C. chrysogaster,* and *C. galeritus* (PP = 1.00; BV = 69%; Fig. 4). The two *C. sanjei* populations were, however, reciprocally monophyletic with respect to other central/East African mangabeys: *C. chrysogaster, C. agilis,* and *C. galeritus* (PP = 1.00; BV = 69%). In this group, *C. agilis* and *C. chrysogaster* formed a group distinct from *C. galeritus* (PP = 1.00; BV = 99%). The concatenated mitochondrial phylogeny showed strong support for the grouping of *C. sanjei* in a group that included the central/East African mangabeys and *M. leucophaeus* (PP = 0.99; BV = 64%; Supplementary material Fig. S2a). Furthermore, the *C. sanjei* populations formed a distinct lineage from the others within this group. No reciprocal monophyly was evident between *Mandrillus* and *Cercocebus*, with *M. leucophaeus* clustering with the four central/East African species. The concatenated nuclear phylogeny based on Bayesian methods showed strong support for the grouping of *C. sanjei* with the central/East African mangabeys (PP = 0.98; BV = 54%, Supplementary material Fig. S2b). The concatenated nuclear phylogeny also confirmed the *Cercocebus* group with strong support based on Bayesian analyses (PP = 0.98; BV = 54%) and showed reciprocal monophyly between *Mandrillus* and *Cercocebus* (PP = 0.98; BV = 54%; Supplementary material Fig. S2b).

### Divergence Time Estimation

**Fig. 5:**
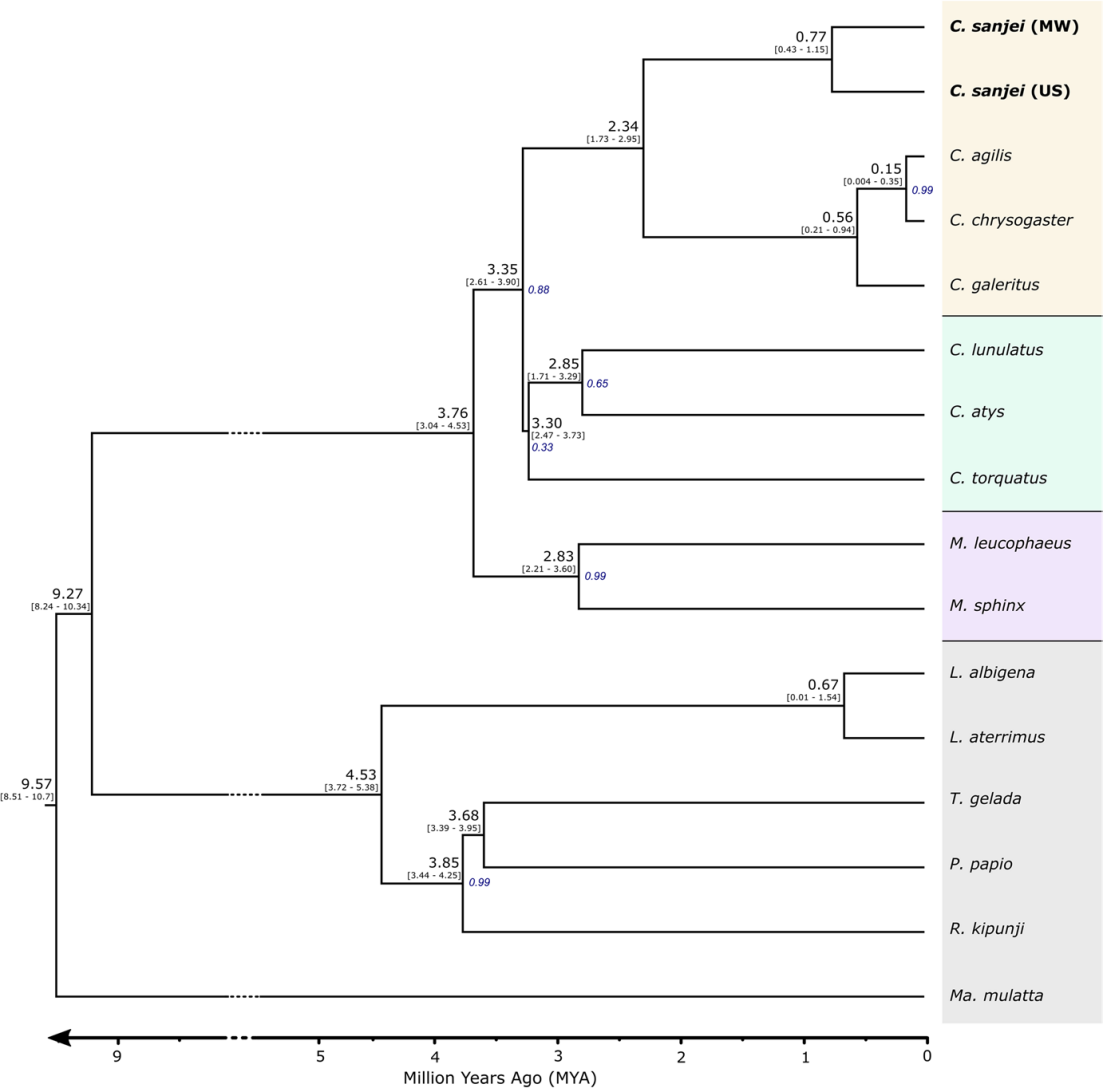
Estimated divergence time of the Sanje mangabey (*Cercocebus sanjei*), Udzungwa Mountains, Tanzania (data collected between June and November 2017). Estimation of divergence time in million years ago (MYA) for papionin species from a concatenated sequence from four genes (CD4, TSPY, and mitochondrial COII and control region; 1,833 base pairs). Estimated time to the most recent common ancestor (TMRCA) is shown at the nodes with the 95% confidence interval underneath in brackets. Posterior probabilities < 1.00 are shown in italics to the right of the corresponding node. Sequences used in the divergence tree are available in Table S2, supplementary material. *Cercocebus* species in Central/East Africa, each previously considered subspecies of *Cercocebus galeritus,* are coloured in yellow; the remaining *Cercocebus* species, found in Central/West Africa, are coloured green; *Mandrillus* species coloured purple; and other Papionin species coloured in grey.

Using the concatenated sequence of all four fragments (CD4, TSPY, COII, and CR; 1,833 bp) in a Calibrated Yule Model, we estimated that the central/East African *Cercocebus* mangabeys (*C. sanjei*, *C. agilis*, *C. chrysogaster,* and *C. galeritus*) and the central/West African mangabeys (*C. lunulatus, C. atys,* and *C. torquatus*) diverged 3.35 MYA (95% Highest Posterior Density (HPD) interval: 2.61–3.90; Fig. 5). We estimated that the *C. sanjei* lineages diverged from *C. agilis*, *C. chrysogaster,* and *C. galeritus* 2.34 MYA (95% HPD interval: 1.73–2.95) and the *C. sanjei* populations diverged from their most recent common ancestor 0.77 MYA (95% HPD interval: 0.43–1.15).

## Discussion

The results of our phylogenetic analyses, with TSPY, CD4, COII, and CR markers concatenated, grouped the Sanje mangabey with the central/East African mangabeys (*Cercocebus* *agilis*, *C. galeritus,* and *C. chrysogaster*), separate from the central/West African mangabeys (*C. torquatus*, *C. lunulatus,* and *C. atys*) with confidence. We estimated the time of divergence for the central/East African mangabeys at 2.34 million years ago (MYA, 95% confidence interval [CI] 1.73–2.95) and a more recent divergence time within this clade for the *Cercocebus agilis*, *C. galeritus,* and *C. chrysogaster* group at ~0.56 MYA (95% CI 0.21–0.94). We estimated a divergence time for the two Sanje mangabey populations at 0.77 MY (95% CI 0.43–1.15). These results suggest that the Sanje mangabey is an evolutionary highly distinct lineage in the *Cercocebus* clade and that the sub-populations are genetically distinct.

Successful amplification and sequencing of nuclear fragments longer than ~350 bp from fecal DNA is typically challenging (Beja Pereira *et al.*, 2009), and our results are consistent with this expectation given the size of the nuclear fragments that we used are larger than that size (i.e., CD4: 400 bp and TSPY: 588 bp). The greater sequencing success for CR compared with the three other markers introduces sampling bias as we investigated the diversity of the other markers in far fewer individuals, who may not represent the diversity of the population accurately.

The group of central/East African taxa that we distinguished included sequences from each of the mangabeys previously considered as subspecies of *Cercocebus* *galeritus* (i.e., *C. sanjei*, *C. galeritus*, *C. agilis,* and *C. chrysogaster*). The nuclear/mitochondrial concatenated phylogeny showed that 1) the central/East African species (i.e., *C. agilis*, *C. galeritus,* and *C. chrysogaster*) share a common ancestor; 2) the central/West African (i.e., *C. torquatus*, *C. lunulatus,* and *C. atys*) share a common ancestor, and 3) *C. torquatus* is a distinct lineage from these groups (Figs. 4 and 5). Our results recapitulate phylogenetic (Guevara *et al.*, 2021) and craniodental studies (Devreese & Gilbert, 2015) that also that suggest *C. agilis* and *C. chrysogaster* have a common ancestor, in a clade including *C. atys*, separated from the *C. torquatus* branch (Supplementary material Table S1, Fig. S1). Additionally, our results support molecular, morphological, and behavioural evidence that suggest a close phylogenetic relationship among the Sanje mangabey, *C. galeritus*, *C. agilis,* and *C. chrysogaster* (Devreese & Gilbert, 2015; Groves, 2001; Grubb *et al.*, 2003; Homewood & Rodgers, 1981; Kingdon, 1997).

**Fig. 6:**
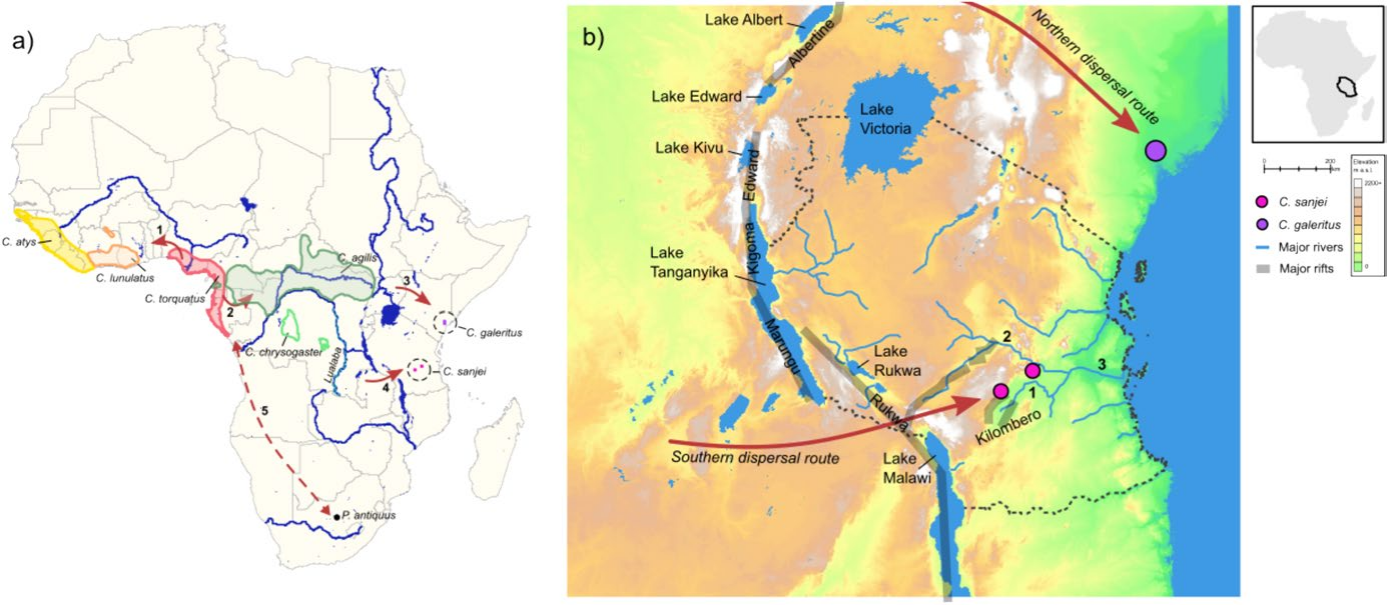
Potential dispersal routes for the Sanje mangabey (*Cercocebus sanjei*) to eastern Africa and potential barriers to dispersal: **a**) the distribution of *Cercocebus* species across Africa with the arrows representing the dispersal from *C. torquatus* west (1) and east (2), creating an eastern and western clades, and the potential two dispersal events for *C. galeritus* (3) and *C. sanjei* (4). The distribution of *C. galeritus* and *C. sanjei* are highlighted by a dashed circle outline and proposed dispersal route either north from the fossil location *P. antiquus* to *C. torquatus* or south from *C. torquatus* to *P. antiquus* (5); and **b**) an elevation map of Tanzania and the surrounding regions, including the locations of the major water bodies, rivers and rifts in Tanzania. Three major rivers surround the Sanje mangabey distribution: Kilombero (1), Ruaha (2), and Rufiji (3). Directionality of dispersal is based on the scenario in the craniodental study by Devreese & Gilbert (2015) that is deemed most representative of the results in this study.

The estimated divergence of central/East and central/West African mangabeys at ~2.34 MYA (95% CI 1.73–2.95) coincides with the estimated dates of a *Procercocebus antiquus* fossil discovered in South Africa (2.0–3.0 MYA, Devreese & Gilbert, 2015). The fossil shows similarities to extant *Cercocebus* mangabeys, with the most craniodental morphological proximity to *C. torquatus* from central/West Africa (Gilbert, 2007). *Cercocebus* species have been hypothesised to have originated in central/West Africa, with the three scenarios suggested by Devreese & Gilbert (2015) showing an eastern dispersal from either of these distributions towards the current central/East African mangabeys (Fig. 6). The fact that *P.* *antiquus* fossil was found in South Africa (Devreese & Gilbert, 2015) raises questions to whether *P. antiquus* represents an early dispersal route from western equatorial Africa to the south or if *Cercocebus* originated in the South and migrated North to diversify into the current *Cercocebus* distributions across Africa (Devreese & Gilbert, 2015)_._ The discovery of more fossils and studies using a greater number of genome-wide nuclear loci to test for consistent monophyly in nuclear phylogenies are required to address these questions.

Within the central/East clade, we estimated a TMRCA of ~2.34 MYA (95% CI 1.73–2.95) between the Sanje mangabey and the *Cercocebus* *galeritus*, *C. agilis,* and *C. chrysogaster*, which is considerably older than the estimated TMRCA of 0.56 MYA (95% CI 0.21–0.94) for these three *Cercocebus* species (Fig. 5). These results suggest that the Sanje mangabey is an evolutionary highly distinct lineage within the *Cercocebus* clade. Our estimate of the approximate divergence time of the Sanje mangabey from other *Cercocebus* mangabeys is very similar to that observed in the mitochondrial divergence found for the sympatric Angolan colobus (*Colobus angolensis palliatus*) population in the Udzungwa Mountains in East Africa and the Guereza colobus (*Colobus guereza*), which has a shared distribution with *C. agilis* across most of central equatorial Africa (TMRCA at ~2.1 MYA, Ting, 2008). As sympatric species, it is likely that the Angolan colobus and the Sanje mangabey experienced similar patterns of dispersal. This coincidence in estimated times of divergence for another primate species inhabiting the Udzungwa Mountains obtained in an independent study provides further support for our estimated divergence times for the Sanje mangabey lineage.

There are three potential explanations for the divergence estimate of the Sanje mangabey from other *Cercocebus* spp. at around 2.34 MYA (Fig. 6). First, the climate in East Africa throughout the Miocene to mid-Pliocene (3–8 MYA) consisted of much warmer and wetter conditions, resulting in an abundance of forest throughout the region (Butynski & Jong, 2020). The following Pliocene-Pleistocene eras consisted of drier periods coinciding with glacial cycles at 3.2, 3.0, 2.8, 1.7, and 1.0 MYA, with a gradual shift of vegetation from closed forest to grassland 3.6 to 2.4 MYA and a significant increase in species adapted to drier environments, 2.8 to 2.4 MYA (deMenocal, 2004). Additionally, from 2.5 MYA onwards, there was an estimated increase in mammal species adapted to grasslands, with the appearance of *Homo erectus* (a *Homo* species thought to be adapted to dry environments) linked to this grassland expansion after 2 MYA (Bobe & Behrensmeyer, 2004). The expansion and contraction of forests and the reduced suitable closed forest habitat available after 2.4 MYA may have influenced dispersal routes between forest fragments, leading to isolation and subsequent speciation. Endemism in the Eastern Arc Mountains has been linked with the stability of ecosystem refuges during periods of climatic change (Fjeldsaå & Lovett, 1997; Marchant *et al.*, 2007; Mumbi *et al.*, 2008), suggesting that ancestral populations of Sanje mangabey may have become isolated in the forests of the Eastern Arc Mountains during these climatic cycles in stable populations.

Second, the proximity of the Sanje mangabey to the Ugalla-Malagarasi and Ruaha-Rufiji rivers suggests that these rivers may have acted as a dispersal barrier, favouring dispersal across the Southern Highlands (Zinner *et al.*, 2015) (Fig. 6b). This pattern has been found for the southern limit for colobines and the Zanzibar dwarf galago (*Paragalago* *zanzibaricus*) in coastal eastern Africa and as the northern limit for the Mozambique dwarf galago (*Paragalago granti*) (Wieczkowski & Butynski, 2013). Additionally, the rivers may have acted as a barrier for the divergence of the northern and southern baboon lineages (*Papio* sp.) around ~2.1 MYA (Kano, 1971; Zinner et al., 2009), coinciding with the divergence time of the Sanje mangabey from other *Cercocebus* mangabeys. Furthermore, the Albertine Rift may have influenced the evolutionary history of the central/East African mangabeys. Significant tectonic activity is estimated to have occurred 2–3 MYA, with the Gregory Rift (northern Kenya) and the Albertine Rift (western Tanzania) both having major uplift events during this time (Partridge *et al.*, 1995; Fig. 6b). It is also estimated that in the last 3 MY there has been active rifting across the Rukwa, Albertine, and Kigoma basins and rift propagation from Lake Malawi and Lake Tanganyika in a southerly and easterly direction (Macgregor, 2015). A paleolake once extended across the western side of the rift, between the current Lake Albert and Lake Edward 2.5–7 MYA and the uplift in this region occurring in the last 2 MY, forming the “rift shoulder” 2.3–2.6 MYA (Fig. 6b; Macgregor, 2015) and the Rwenzori Mountains 2 MYA (Kaufmann *et al.*, 2016).

Third, intraspecific competition may have influenced dispersal and isolation alongside physical barriers. The current and ancient widespread *Cercopithecus* species are thought to have influenced the relictual distribution of *Cercocebus* species through competition (Wieczkowski & Butynski, 2013). The historic dispersal of *Cercopithecus* sp. in East Africa is thought to have also been influenced by the Rift Valley and increasing unsuitable arid habitat (Fjeldsaå & Lovett, 1997). The expansion of *Cercopithecus* (*nictitans*) species diversity is estimated to have occurred 2.1–2.4 MYA (Fjeldsaå & Lovett, 1997), coinciding with the divergence (2.34 MYA) of the Sanje mangabey from other *Cercocebus* species. This diversification and the expansion and contraction of forest habitats may have led to direct competition between the ancestral central/Eastern *Cercocebus* populations with *Cercopithecus* sp. (e.g., ancestral populations of *Cercopithecus mitis* found in the Udzungwa Mountains), resulting in the isolation of populations, prevention of dispersal between contracting intermediate forests, and possibly leading to the extinction of *Cercocebus* populations. It is also likely that the Sanje mangabey experienced competition with other semiterrestrial primates or were isolated due to avoidance of other species, such as the yellow baboon (*Papio* *cynocephalus*), that are well-adapted to the lower elevation forests and savannahs. Currently, mangabeys avoid interactions with baboons (McCabe & Fernández, personal communication). Tana River mangabeys, endemic to southeastern Kenya, have also been observed avoiding yellow baboons in regions used by the two species, reducing their within-group dispersal when habitat overlap was particularly high with baboons and being displaced in sleeping sites (Wahungu, 1998). These behaviors may reduce habitat overlap between the species and lead to lower competition. However, the behaviors were observed in a period of high fruit availability, which reduced the need for overlap with abundant resources available across the habitat area (Wahungu, 1998). In periods of reduced food resources, both species may occupy the same habitats, potentially increasing competition and likely to the detriment of the mangabeys (Wahungu, 1998). Therefore, it is possible that avoidance and competition for resources between mangabeys and other species limited dispersal between forest habitats at a time the forests were becoming smaller and fewer, isolating Sanje mangabey populations.

Our findings for *Cercocebus* sp., match phylogenies of the central/East African red colobus (*Piliocolobus* sp.) species, which are also divided into two clades: one grouping the Kenyan Tana River colobus (*P. rufomitratus*; sympatric with *C. galeritus*) with central African colobines, and one grouping the Udzungwa (*P. gordonorum*: sympatric with the Sanje mangabey) and Zanzibar red colobus (*P. kirkii*; Ting, 2008). A study of the cranial morphology of red colobus species suggested distinct morphology in *P. rufromitratus*, more closely resembling ancestral central species than the Udzungwa and Zanzibar colobines, pointing to two dispersal events into East Africa (Cardini & Elton, 2009). The pattern that we found for the central/East African *Cercocebus* mangabeys resembles this pattern. Cardini & Elton (2009) suggest the ancestral Tana River red colobus dispersed North of the Rift Valley and the Lualaba River (Fig. 6a), through montane refugia across to the Tana forests. The Udzungwa and Zanzibar red colobines dispersed along a southern route from Lake Tanganyika across the Southern Highlands to the Udzungwas and the forests along the Ruaha-Rufiji River, as described for *Cercopithecus* species (Butynski & Jong, 2020). Devreese & Gilbert (2015) suggested that *C.* *galeritus* and *C. sanjei* became relicts recently. However, our study supports their alternative hypothesis that each species arrived at their current distribution through separate dispersal events following that of *Piliocolobus* sp. (Devreese & Gilbert, 2015; Ting, 2008)*.* Overall, these results suggest the ancestral Sanje mangabey may have followed the southern dispersal route away from the other central/East African mangabeys (Fig. 6b).

We estimated the TMRCA for the two Sanje mangabey populations at 0.77 MY (95% CI 0.43–1.15). Since we did not find differences in TSPY nuclear sequences or the mitochondrial COI sequence, this divergence time based only on differences in the nuclear CD4 and mitochondrial CR fragment. A previous estimate suggested that the forests separated more than 100 years ago (Struhsaker *et al.*, 2004); however, our estimation of TMRCA for the two populations alongside other studies of population genetic structure in the Udzungwa Mountains forests (East Afromontane horned chameleons, *Triceros* sp., Ceccarelli *et al.*, 2014, and grey-faced sengi, *Rhynchocyon udzungwensis*, Lawson *et al.*, 2013), suggests that the division of subpopulations across taxa within the forest fragments occurred over a much more ancient timescale. The divergence time we found suggests the two populations of Sanje mangabey represent two ancient lineages, rather than becoming isolated with more recent anthropogenic activity.

Our study suggests that the isolation of the Sanje mangabey from other *Cercocebus* mangabeys followed an ancient isolation of the two populations to relatively distant (~100 km) forest blocks, potentially as the result of a drying climate in the region and physical barriers, coupled with interspecific competition and avoidance from other terrestrial primate species preventing dispersal between forest blocks. In another study that aimed to investigate the phylogeographic history of the Sanje mangabey and recent changes in size and range of the two populations, we explored whether lack of suitable habitat across the past 40,000 years could have influenced genetic differentiation using a habitat suitability modelling approach (Paddock *et al.*, 2026). We described six haplotypes in 64 mitochondrial control region sequences and significant genetic differentiation among the populations, which also show no recent changes in their size (Paddock *et al.*, 2026). Results from the modelling exercises suggest that a general trend in aridification in East Africa across the past 40,000 years, which resulted in a shift of montane forests to gradually higher elevations, may have limited dispersal and gene flow, potentially leading to the large genetic differentiation found between populations (Paddock *et al.*, 2026). Together, these results suggest that the two subpopulations represent evolutionarily distinct lineages. A larger study of Sanje mangabey population genetic structure using nuclear genetic or genome-wide nuclear would help to support inferences of both recent (up to two generations ago) and historic events.

Our study serves as an example of the need to study population level genetic structure to identify cryptic diversity within species, particularly with conservation efforts in mind. Our results suggests that the Sanje mangabey lineage is evolutionarily distinct from other mangabeys and therefore warrants conservation protection as a distinct management unit. In addition, the two Sanje mangabey populations should be considered candidate ESUs (Moritz, 1994) and conservation management planning must take this into consideration. The estimated time of divergence between populations was on a longer time scale than expected by previous studies (e.g., more than 100 years ago; Struhsaker *et al.*, 2004) and may have important conservation implications. Future morphological, molecular, and behavioural studies are required to determine how reproductive isolation has shaped these features. If conservation actions, such as genetic rescue, translocation, or captive breeding, are to be considered, the risk of outbreeding depression between populations should be estimated prior to action.

## Supplementary Information

Below is the link to the electronic supplementary material.

Supplementary file1 (DOCX 525 KB)

## Data Availability

The datasets generated during or analysed during the current study are available in the GenBank repository withaccession numbers PP896812 to PP896819. Additional data are available in the Supplementary Information.

## Declarations

### Additional Information

Supplementary information accompanies this paper.
