# Supplementary material for: On the Phylogenetic History of the Sanje Mangabey (Cercocebus sanjei), Udzungwa Mountains, Tanzania
Source: Int J Primatol. 2026 Apr 27;47(3):842–67. doi: 10.1007/s10764-026-00559-w (PMC13348350; doi:10.1007/s10764-026-00559-w)
Supplement: Supplementary file 1 — Supplementary file1 (DOCX 525 KB) [file 10764_2026_559_MOESM1_ESM.docx]

*Supplementary material -* On the phylogenetic history of the Sanje mangabey (*Cercocebus sanjei*)

Paddock et al

**Supplementary Table S1** Previous nomenclature used to describe the Sanje mangabey as either species level (*Cercocebus sanjei*) or subspecies level (*Cercocebus galeritus sanjei*) in its first description, systematic reviews including the Mangadrills and in previous IUCN Red List reports. * First description; ** IUCN Red List Assessment

| **Study** |  | **Nomenclature** |
| --- | --- | --- |
| Homewood et al. (1981) * | ^[8]^ | *Cercocebus galeritus sanjei* |
| Mittermeier (1986) |  | *Cercocebus galeritus sanjei* |
| Groves (1996) | ^[75]^ | *Cercocebus sanjei* |
| Kingdon (1997) | ^[5]^ | *Cercocebus sanjei* |
| Butynski et al. (2000) ** | ^[76]^ | *Cercocebus galeritus sanjei* |
| Groves (2001) | ^[15]^ | *Cercocebus sanjei* |
| Grubb et al. (2003) | ^[13]^ | *Cercocebus galeritus sanjei* |
| Groves (2005) | ^[77]^ | *Cercocebus sanjei* |
| Ehardt et al. (2008) ** | ^[78]^ | *Cercocebus galeritus sanjei* |
| Mittermeier et al. (2013) | ^[14]^ | *Cercocebus sanjei* |
| McCabe et al. (2019) ** | ^[7]^ | *Cercocebus sanjei* |

**Section 1. Past morphological and genetic studies on the *Cercocebus* species**

A number of morphological studies and several using nuclear DNA have determined that the *Cercocebus* species can be divided into two groups. The Central/West African species (Sooty mangabey *C. atys*, white-naped mangabey *C. lunulatus*, and the collared mangabey *C. torquatus*) are broadly distinguishable from species in Central/East Africa (Tana River mangabey *C. galeritus,* golden-bellied mangabey *C. chrysogaster* and the agile mangabey: *C. agilis* (Davenport et al., 2006; Devreese & Gilbert, 2015; Disotell, Honeycutt, & Ruvolo, 1992; Guevara & Steiper, 2014), (Supplementary Figure S1). Other studies, including those using mitochondrial DNA, suggest paraphyly between *Cercocebus* and *Mandrillus* lineages (Davenport et al., 2006; Liedigk et al., 2014; Van der Kuyl et al., 1995; Zinner et al., 2011), or nuclear studies that showed no distinct clades within the genera (e.g., α 1,3 GT marker, Davenport et al. (2006); Disotell et al. (1992); TSPY marker: Disotell et al. (1992) in Supplementary Figure S1). No phylogenetic study performed to date has included molecular sequence data from all species (Figure S1) so questions about relationships and lineage distinctiveness remain. Given that eight of nine species within the *Cercocebus-Mandrillus* clade are under threat of extinction (two are considered Vulnerable, five are Endangered, and one is Critically Endangered: <https://www.iucnredlist.org>; Figure 3), establishing the phylogenetic relationships within this clade and of populations within each lineage is essential for effective conservation.

|  |
| --- |
| 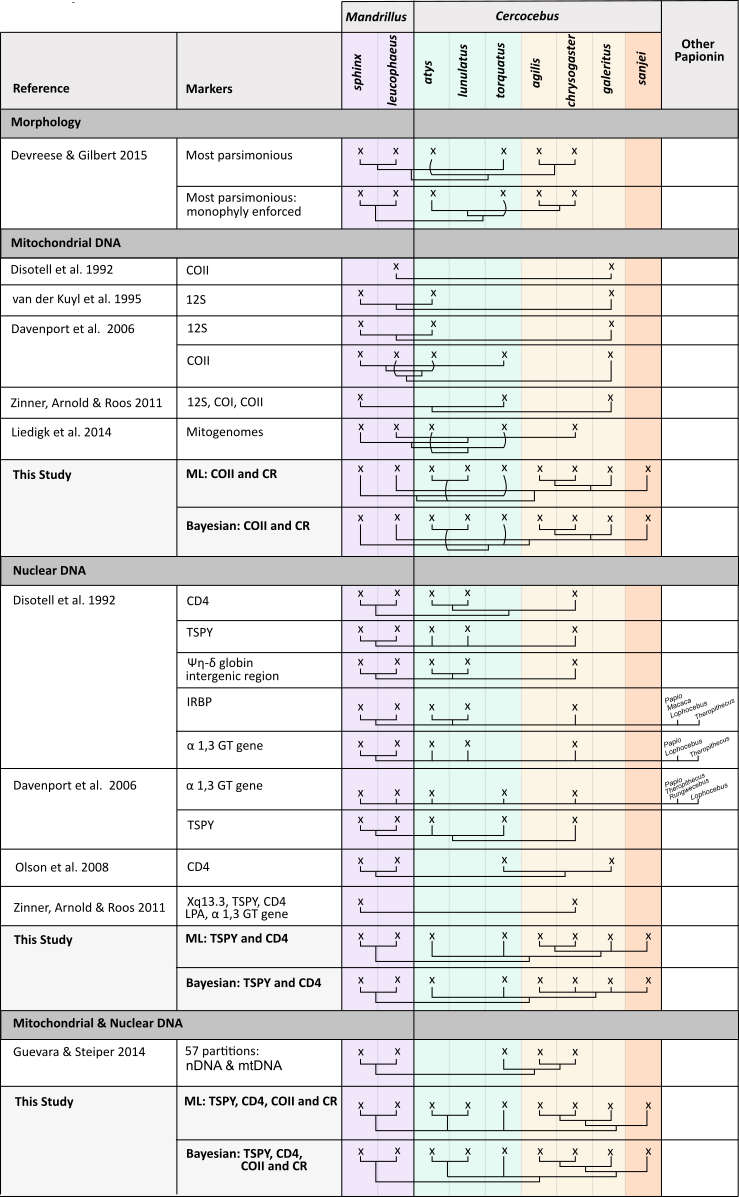 |

**Figure S1.** A compilation of the most recent craniodental morphological study by Devreese & Gilbert (2015) and the past phylogenetic studies that included at least one *Cercocebus* and one *Mandrillus* species for comparison to the phylogenies from this study.

| **Supplementary Table S2.** GenBank accession numbers for sequences used in this study: one autosomal marker (CD4), one nuclear Y-chromosome marker (TSPY), and two mitochondrial genes; control region (CR) and cytochrome oxidase subunit II (COII). ^a^Used in nuclear and full concatenated sequence; ^b^Used in mtDNA and full concatenated sequence | | | | |
| --- | --- | --- | --- | --- |
|  | **CD4** | **TSPY** | **COII** | **CR** |
| *Cercocebus sanjei* | Generated in this study | | | |
| *Cercocebus galeritus* |  | AY195576^a^ | AY686132^b^  M74004 |  |
| *Cercocebus agilis* | FJ750597 ^a^ | FJ560633 ^a^ | FJ750650 ^b^ |  |
| *Cercocebus chrysogaster* | AF057382 ^a^ | AF057410 ^a^ | NC021943 ^b^ | NC021943 ^b^ |
| *Cercocebus lunulatus* |  |  | AY686134 ^b^ |  |
| *Cercocebus torquatus* |  | AY195577 ^a^ | NC023964 ^b^  AY686133  FJ713422 | NC023964 ^b^ |
| *Cercocebus atys* | AF057383^a^  AF057384 | AF057411  AF057412  AF057413 ^a^ | AY686135  KP090062  KT159932  NC028592 ^b^ | KP090062  NC028592 ^b^ |
| *Mandrillus sphinx* | AF057386 ^a^  FJ750596 | FJ750632 ^a^ | FJ713421 ^b^  FJ750651 | KC757403 ^b^  KJ434963 |
| *Mandrillus leucophaeus* | AF057387 ^a^ | AF057421 ^a^ | M74006 ^b^ | KT696596 ^b^ |
| *Lophocebus aterrimus* | AF057390 ^a^  FJ750595 | AF057423 ^a^  FJ750631 | FJ713415 ^b^ |  |
| *Lophocebus albigena* | AF057391 ^a^ | AF057425 ^a^ |  |  |
| *Theropithecus gelada* | AF057389 ^a^  FJ750594 | AF057415 ^a^ | EU293079 ^b^  M74009 | NC019802 ^b^ |
| *Rungwecebus kipunji* | EU600174 ^a^ | DQ381472 ^a^ | DQ381471 ^b^ |  |
| *Papio papio* | FJ750590 ^a^  AF057388 | FJ750626 ^a^ | EU293078 ^b^ | NC020009 ^b^ |
| *Macaca mulatta* | AF057385 ^a^ | AF057416 ^a^  AF425276 | M74005 ^b^ | KJ567051 ^b^ |
|  | | | | |

| **Supplementary Table S3**. Number of fecal samples collected per sampling site, number of sequences obtained per genetic marker and size of fragment in base pairs (bp) - autosomal CD4 gene (CD4); Y chromosomal testis-specific protein (TSPY), cytochrome oxidase subunit II (COII), mitochondrial control region (CR). The number of haplotypes found (hap) is shown. | | | | | |
| --- | --- | --- | --- | --- | --- |
|  |  | | | | |
| Populations | Samples collected | Sequences obtained: number of haplotypes | | | |
|  |  | CD4 (400 bp) | TSPY (588 bp) | COII (480 bp) | CR (369 bp) |
| Mwanihana Forest | 117 | 4: 1 hap | 2: 1 hap | 4: 1 hap | 36: 2 hap |
| Uzungwa Scarp Nature Reserve | 56 | 2: 1 hap | 4: 1 hap | 8: 1 hap | 28: 4 hap |
| Total | 173 | 6: 1 hap | 6: 1 hap | 12: 1 hap | 64: 6 hap |

| **Supplementary Figure 2.** Phylogenetic trees for Papionin species using two concatenated sequences: a) mitochondrial cytochrome c oxidase subunit II (COII) and control region concatenated (849 bp) and b) nuclear CD4-TSPY concatenated (988 bp). Both the Maximum Likelihood phylogeny (left of species names; bootstrap values shown at the nodes) and the Bayesian phylogeny (right of the species names; posterior probabilities shown at the nodes) are presented. Sequences used in the phylogeny are available in Supplementary Table 2. Cercocebus species in Central/East Africa, each previously considered subspecies of Cercocebus galeritus, are coloured in yellow, the remaining Cercocebus species, found in Central/West Africa, are coloured green, Mandrillus species coloured purple and other Papionin species coloured in grey. | |
| --- | --- |
| 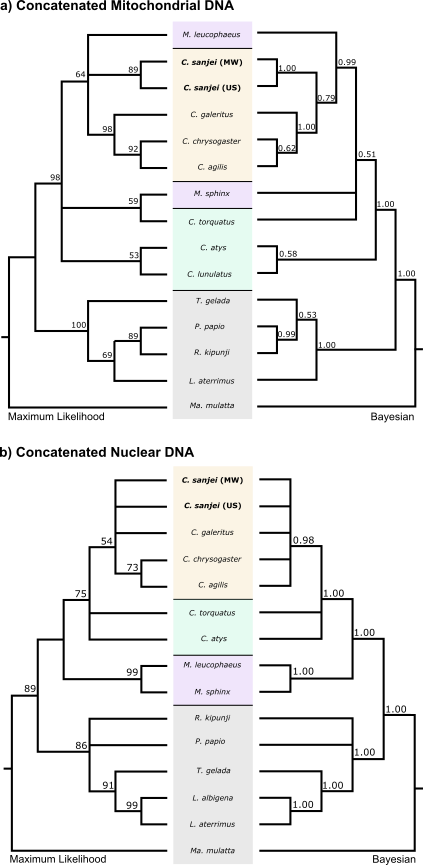 |  |

**Section 2. Considerations on the phylogenetic relation between *Cercocebus* and *Mandrillus* as inferred by the concatenated mitochondrial phylogeny**

In the concatenated mitochondrial phylogeny, *Cercocebus* species were paraphyletic with *Mandrillus,* however *Cercocebus* and *Mandrillus* were monophyletic in the concatenated nuclear tree. Incongruence between nuclear and mitochondrial phylogenies can indicate either incomplete lineage sorting or ancient hybridisation and introgression (Petit & Excoffier, 2009). Previous studies of mitochondrial phylogeny (Liedigk et al., 2014; van der Kuyl et al., 1995), and molecular (Baba et al., 1980; Dene et al., 1976; Hewett-Emmett et al., 1976; Stanyon et al., 1988), morphological (Devreese & Gilbert, 2015; Fleagle & McGraw, 2002) and behavioural similarities (Groves, 1978) have also indicated a very close relationship between the *Cercocebus* and *Mandrillus* species. Considering previous studies of nuclear and mitochondrial DNA (Davenport et al., 2006; Guevara et al., 2021; Liedigk et al., 2014; Perelman et al., 2011; Zinner et al., 2009, 2011), Devreese & Gilbert (2015) suggested it may be possible that an ancient introgression event occurred with the overlap of the range of *Cercocebus* and *Mandrillus* in western Africa before the dispersal events out from this region towards east Africa. To further understand the relationship between the *Cercocebus* and *Mandrillus* species it would be beneficial to undertake extensive sampling from across the current known distribution for each species. This would identify whether isolated populations hold cryptic diversity that may provide insight into the dispersal routes by identifying time to the most recent common ancestor at both the population and species level. This study also used only four markers, therefore, genome-wide markers combined with behavioural and morphological studies would provide a more accurate estimate of relationships between species.

**References**

Baba, M., Darga, L. and Goodman, M. (1980). Biochemical Evidence on the Phylogeny of Anthropoidea. In: Ciochon, R. L. and Chiarelli, A. B. (eds.) Evolutionary Biology of the New World Monkeys and Continental Drift. Advances in Primatology. Boston, MA: Springer.

Dene, H.T., Goodman, M. and Prychodko, W. (1976). Immunodiffusion evidence on the phylogeny of the primates. In: Goodman, M. and Tashian, R. E. (eds.) Molecular Anthropology. New York: Plenum, pp. 171–195.

Davenport, T.R.B., Stanley, W.T., Sargis, E.J., De Luca, D.W., Mpunga, N.E., Machaga, S.J. and Olson, L.E. (2006). A new genus of African monkey, Rungwecebus: Morphology, ecology, and molecular phylogenetics. Science [Online] 312:1378–1381. Available at: http://www.sciencemag.org/cgi/doi/10.1126/science.1125631.

Devreese, L. and Gilbert, C.C. (2015). Phylogenetic relationships within the Cercocebus-Mandrillus clade 147as indicated by craniodental morphology: Implications for evolutionary biogeography. American Journal of Physical Anthropology [Online] 158:227–241. Available at: http://doi.wiley.com/10.1002/ajpa.22780.

Disotell, T.R., Honeycutt, R.L. and Ruvolo, M. (1992). Mitochondrial DNA phylogeny of the Old-World monkey tribe Papionini. Molecular Biology and Evolution 9:1–13.

Fleagle, J.G. and McGraw, W.S. (2002). Skeletal and dental morphology of African papionins: unmasking a cryptic clade. Journal of Human Evolution [Online] 42:267–292. Available at: https://linkinghub.elsevier.com/retrieve/pii/S0047248401905263.

Groves, C.P. (1978). Phylogenetic and population systematics of the Mangabeys (primates: Cercopithecoidea). Primates 19:1–34.

Guevara, E.E. and Steiper, M.E. (2014). Molecular phylogenetic analysis of the Papionina using concatenation and species tree methods. Journal of Human Evolution [Online] 66:18–28. Available at: http://dx.doi.org/10.1016/j.jhevol.2013.09.003.

Guevara, E.E. and Steiper, M.E. (2014). Molecular phylogenetic analysis of the Papionina using concatenation and species tree methods. Journal of Human Evolution [Online] 66:18–28. Available at: http://dx.doi.org/10.1016/j.jhevol.2013.09.003.

Hewett-Emmett, D., Cook, C.N. and Barnicott, N.A. (1976). Old World monkey hemo-globins: deciphering phylogeny from complex patterns of molecular evolution. In: Goodman, M. and Tashian, R. E. (eds.) Molecular Anthropology. New York: Plenum, pp. 257–275.

Liedigk, R., Roos, C., Brameier, M. and Zinner, D. (2014). Mitogenomics of the Old World monkey tribe Papionini. BMC Evolutionary Biology [Online] 14:176. Available at: [http://dx.doi.org/10.1186/s12862- 014-0176-1](http://dx.doi.org/10.1186/s12862-%20014-0176-1).

Petit, R.J. and Excoffier, L. (2009). Gene flow and species delimitation. Trends in Ecology and Evolution 24:386–393.

Perelman, P., Johnson, W.E., Roos, C., Seuanez, H.N., Horvath, J.E., Moreira, M.A.M., Kessing, B., et al. (2011). A molecular phylogeny of living primates. PLoS Genetics 7:1–17.

Van Der Kuyl, A.C., Kuiken, C.L., Dekker, J.T. and Goudsmit, J. (1995). Phylogeny of African monkeys based upon mitochondrial 12S rRNA sequences. Journal of Molecular Biology 40:173–180.

Stanyon, R., Fantini, C., Camperio‐Ciani, A., Chiarelli, B. and Ardito, G. (1988). Banded karyotypes of 20 Papionini species reveal no necessary correlation with speciation. American Journal of Primatology 16:3–17

Zinner, D., Arnold, M.L. and Roos, C. (2011). The strange blood: Natural hybridization in primates.

Evolutionary Anthropology 20:96–103.
